# C-type lectins in HIV-1 infection

**DOI:** 10.1186/1742-4690-8-S2-O27

**Published:** 2011-10-03

**Authors:** Teunis BH Geijtenbeek

**Affiliations:** 1 Host Defense group, Center for Experimental and Molecular Medicine, Academic Medical Center, Amsterdam, The Netherlands

## 

Adaptive immune responses by dendritic cells (DCs) are controlled by pattern recognition receptors such as Toll-like receptors (TLRs) and C-type lectins. C-type lectins interact with carbohydrate structures on pathogens. Upon pathogen binding, C-type lectins trigger signaling pathways that induce specific cytokines to dictate T cell polarization. Thus, C-type lectins are crucial in tailoring immune responses to pathogens. HIV-1 is recognized by the C-type lectin DC-SIGN. Previous data have shown that DC-SIGN is involved in HIV-1 transmission by DCs. DCs efficiently capture HIV-1 and transmit the virus to T cells. However, recent data show that DC-SIGN also induces signaling that shape adaptive immune responses. Here I will discuss the molecular signaling pathways induced by DC-SIGN that are involved in adaptive immunity to HIV-1. Notably, HIV-1 hijacks the signaling by DC-SIGN and TLR8 for its own replication and transmission. The subversion of these crucial immune signaling pathways by HIV-1 and the consequences for HIV-1 infection will also be discussed.

